# Insights into the microbiome of mine drainage from the Mária mine in Rožňava, Slovakia: a metagenomic approach

**DOI:** 10.3389/fmicb.2025.1675058

**Published:** 2025-11-28

**Authors:** Lenka Hagarová, Daniel Kupka

**Affiliations:** Institute of Geotechnics of the Slovak Academy of Sciences, Kosice, Slovakia

**Keywords:** microbial communities, mine drainage, metagenomics, Slaná River, Mária mine

## Abstract

The Mária mine, particularly the Strieborná vein, in Rožňava, is one of the most important mines in Slovakia, containing Ag-bearing tetrahedrite (40–46 wt% Cu, 26 wt% Sb, ~1 wt% Ag), making it an important source of strategic and critical raw materials. This mine discharges a unique neutral-pH (6.9), metal-rich mine water drainage (402 mg L^−1^ SO_4_^2−^, 4.65 mg L^−1^ Fe) that has remained microbiologically uncharacterized. This study presents the first comprehensive shotgun metagenomic survey of this mine effluent, generating ~227 million high-quality reads that assembled into 157,676 contigs and 378,023 non-redundant genes. Taxonomic analysis revealed a community dominated by Betaproteobacteria (> 66%), with abundant lithotrophic genera *Sulfuritalea* (6.93%), *Ferrigenium* (5.45%), *Gallionella* (3.79%), and *Sideroxydans* (3.65%), alongside the heterotrophic genus *Pseudomonas* (5.2%). Among the most prevalent neutrophilic iron-oxidizing bacterial strains were *Sulfuritalea hydrogenivorans* (6.93%), *Ferrigenium kumadai* (5.45%) and *Gallionella capsiferriformas* (3.79%). Acidophilic genera (e.g., *Thiobacillus* sp. at 0.43%, *Ferrovum myxofaciens*, *Acidithiobacillus ferrivorans*, *Leptospirillum ferrooxidans*) collectively accounted for <1% of the community. Functional annotation against KEGG, CAZy, COG, eggNOG, Swiss-Prot, CARD and BacMet databases demonstrated pronounced enrichment of iron cycling (e.g., the iron complex outer-membrane receptor protein TC.FEV.OM), sulfur oxidation (e.g., SoxA, SoxX, SoxB), carbon turnover (glycosyltransferase and glycoside hydrolase families) and nitrogen cycling (e.g., NifH, NifD, NirK, glnA). The antibiotic-resistance profile was dominated (> 95%) by tetracycline and fluoroquinolone determinants, while metal-resistance systems for Ni, Ag, As, Cu and Zn (including CzcD, CzcA, CznA, ArsD and AioX/AoxX) were likewise pervasive. This integrated taxonomic-functional portrait highlights a microbiome finely adapted to this unique geochemistry, combining lithotrophic metabolisms with multi-metal resistance. Our findings establish a critical baseline for long-term monitoring and highlight a high abundance of neutrophilic Fe(II)-oxidizers, suggesting they may represent promising candidates for targeted cultivation and subsequent evaluation in biotechnology applications.

## 1 Introduction

The Rožňava ore field, located on the southeastern margin of the Western Carpathians, is characterized by siderite-sulfidic veins. The Strieborná vein is notable for its rich deposits of tetrahedrite, chalcopyrite, pyrite, and arsenopyrite (Mikuš et al., 2018). Moreover, massive vein-hosted tetrahedrite areas are enriched in silver at grades of 150–450 g/t (Sasvári and Maťo, 1998). Currently, the Mária mine in Rožňava, holds significant economic interest primarily due to the presence of Ag-bearing natural tetrahedrite (Cu_12_Sb_4_S_13_). This copper-antimony sulfosalt contains a high Cu content (40–46 wt%) and relatively high content of Ag (up to 1 wt%). However, tetrahedrite-group members carry different proportions of arsenic, antimony, mercury, or cadmium. Thus, subsequent exposure of tetrahedrite to oxidizing conditions may result in the mobilization of Sb, As, Cu, S, and other elements present in their structure, contributing to toxicological and environmental risks (Hiller et al., 2013).

In general, acid mine drainage (AMD) results from the oxidative dissolution of sulfide minerals exposed to oxygen and water in ore mine-associated areas (Nordstrom and Alpers, 1999). Although abiotic processes can oxidize sulfide minerals, the rate of reaction is greater by many orders of magnitude in the presence of certain lithotrophic acidophiles, such as iron-oxidizing chemolithotrophs (Johnson and Hallberg, 2003). The role of acidophilic prokaryotes in this process is to oxidize ferrous iron to ferric iron, thereby maintaining a high redox potential defined by the Fe^3+^/Fe^2+^ ratio. For example, *Leptospirillum (L.) ferriphilum* has a high affinity for Fe^2+^ substrate, low sensitivity to the Fe^3+^ product, tolerates lower pH levels of the medium and elevated cultivation temperature, making it a key player in the bio-oxidative leaching of sulfide minerals (Kupka et al., 2023).

Characterization of mine-water microbial consortia and identification of the major iron- and/or sulfur- oxidizing bacteria are essential for the development of effective approaches to prevent and mitigate the impact of AMD (Tan et al., 2009). The structure and composition of bacterial communities are shaped by a complex set of evolutionary, ecological, and environmental factors (Berendonk et al., 2015). The selective factors that shape AMD-associated microbial communities are pH, temperature, high concentrations of dissolved metals and metalloids, total organic carbon (TOC), and dissolved oxygen (DO) (Tan et al., 2009; Kuang et al., 2013). Additionally, AMD typically contains a high level of sulfate, heavy metals such as iron, zinc, copper, cadmium, aluminum, and metalloids like arsenic, whereby may cause serious adverse effect on human health and ecological resources (Rakotonimaro et al., 2017; Măicăneanu et al., 2013).

By applying high-throughput sequencing, our understanding of microbial diversity has been rapidly expanding, which has given more comprehensive insights into the role of microorganisms in their specific niches. Given the enormous and complex nature of microbial diversity, the importance of metagenomics has increased. Next generation technologies (NGS) have proven their utility in shotgun metagenomic since their application in 2006 (Kim et al., 2013). In shotgun metagenomics, the total DNA is extracted and then exposed to random fragmentation before the NGS. This allows extensive characterization of strain-level multi-kingdom taxonomic classification, functional profile characterization, and detection of antimicrobial resistance. In contrast, 16S rRNA sequencing primarily utilizes PCR to amplify a specific gene region (such as V3, V4), which is then templated for NGS, thus generating only 16S gene sequences. Additionally, 16S rRNA microbial community profiling commonly uses an operational taxonomic unit (OUT)-based approach (Sharpton, 2014).

This study presents a comprehensive analysis of the bacterial communities in mine drainage associated with siderite/tetrahedrite-dominated ore at the Mária mine, paired with essential geochemical characterization and with basic functional profiling. As the only known mine drainage, which is located on the periphery of the Rožňava city, the mine effluent impacts the Slaná River, which merges with the Rimava River and subsequently flow into a transboundary waterway in Hungary. Consequently, this work is essential for characterizing local microbial and geochemical profiles and establishes a reference framework to support future assessments of potential cross-border ecological risks.

## 2 Materials and methods

### 2.1 Sample collection and chemical analysis

**Figure 1:**
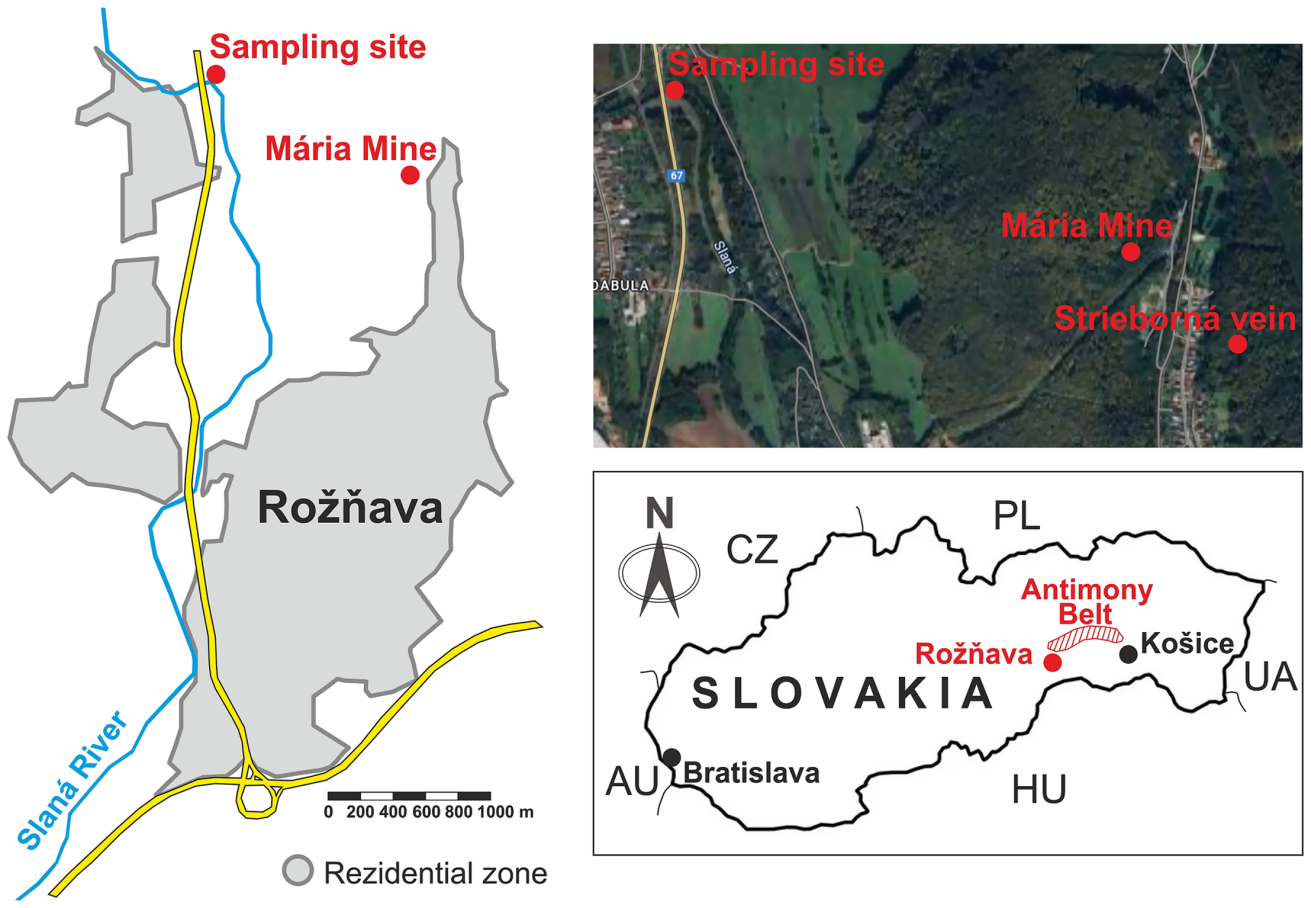
Location of the sampling site and Mária mine in Rožňava, Eastern Slovakia.

The water sample was collected at the end of March 2025 from the outdoor discharge pipe of the Mária mine (Rožňava) (Figure 1). The sampling site is located above ground, fully exposed to natural light and atmospheric conditions, and represents the drainage outlet from an underground tunnel. The site is situated near a road bridge but outside the residential zone of Rožňava. Additional photographs of the site are provided in the Supplementary Figure S1. The water sample was taken using sterile flasks. The sample volume was 10 L. The pH value was measured using a pH meter (Denver Instrument UltraBasic pH Benchtop Meter), temperature and conductivity by conductometer (WTW Cond 330i Set, TetraCon 325). Ion and metal content was determined by ICP spectrometer and ion chromatograph Dionex ICS 5000 (Sunnyvale, CA, United States).

### 2.2 Genomic DNA extraction

The water sample was filtered by a vacuum bottle filter (JET Biofil, China) with a 50 mm diameter polyethersulfone (0.22 μm pore diameter) filter membrane. The filter was then placed into a 15 mL tube with 3 mL TE buffer (10 mmol/L Tris-EDTA, pH 8) and vortexed for 5 min. Total genomic DNA was extracted from bacterial biomass containing TE buffer using QIAamp BiOstic Bacteremia DNA Kit (Qiagen, Germany), optimized for low bacterial content samples. The quality and concentration of DNA were evaluated by a NanoDrop ONE (Thermo Scientific, United States).

### 2.3 Library preparation and sequencing

Extracted DNA was purified using the Agencourt AMPure XP-Medium kit (Beckman Coulter, United States) and a library of DNA fragments of ~300 bp in length was constructed. The purified DNA fragments were detected using the Qubit dsDNA HS Assay Kit and sheared to the target size using the Covaris ultrasonicator (Covaris, United States). The DNA fragment ends were aligned, phosphorylated and 3′-adenylated followed by ligation of adapters. PCR amplification of the ligated products and purification using the Agencourt AMPure XP-Medium kit were carried out. In the next step, the PCR products were detected using the 2,100 Bioanalyzer (Agilent, United States). The double-stranded PCR products were denatured and circularized by split ligation with the gain of single stranded circular PCR products. After removal of single stranded linear products, the final form of DNA library was obtained. The concentration and length of the fragments were checked using the 2,100 Bioanalyzer (Agilent, United States). The final DNA library was sequenced using the DNBSEQ PE150 (BGI Tech Solutions (Hong Kong) Co., Ltd., Hong Kong, China) platform.

### 2.4 Read quality control and assembly

Raw data were subjected to quality control with exclusion of reads containing 10% uncertain bases (N bases), reads containing adapter sequences (15 bases or longer sequence aligned to the adapter sequence), and reads with low quality bases (Q < 20%). Raw sequencing data were filtered using SOAPnuke v1.5.0 (Chen et al., 2018) and decontaminated of human sequences via Bowtie2 (Langmead and Salzberg, 2012) alignment. The cleaned data, i.e., high-quality short reads, were *de novo* assembled into contigs using the MEGAHIT v1.1.3 (Li et al., 2015) (k-mer range 33–53, min contig length = 200 bp).

### 2.5 Gene prediction and catalogue construction

Prediction of structural genes was performed using MetaGeneMark 3.38 (Zhu et al., 2010) to find open reading frames (ORFs) from each contig. The CD-HIT v4.6.4 (Fu et al., 2012) clustering algorithm was used to generate a non-redundant gene set, which sorts the sequences in descending order of length.

### 2.6 Taxonomic profiling

High-quality reads were classified with Kraken2 v2.1.2 (Wood et al., 2019) against the NCBI NT database with species level abundance refined by Bracken v2.6.2 via KrakenTools v1.2. Taxonomic annotation was also carried out against UHGG v2 database (Almeida et al., 2021).

### 2.7 Functional annotation of gene catalogue

Non-redundant genes were annotated by DIAMOND v.2.0.11 (Buchfink et al., 2015) in BLASTP using parameters: parameters:--evalue 1e-5 --threads 2 --outfmt 6 –seg no --max-target-seqs 20 --more-sensitive -b 0.5 –salltitles against databases: KEGG (v109) (Ogata et al., 1999), CAZy (20211013) (Lombard et al., 2014), COG (20201125) (Galperin et al., 2015), eggNOG (v5.0) (Huerta-Cepas et al., 2019), Swiss-Prot (release-2024_01) (Poux et al., 2017), CARD (v3.0.9) (Jia et al., 2017), and BacMet (20180311) (Pal et al., 2014).

### 2.8 Statistical analyses

To assess overall community structure differences in species and functional composition, a Permutational Multivariate Analysis of Variance (PERMOVA) was applied to Principal Coordinates Analysis (PCoA) distance matrices.

## 3 Results

### 3.1 Geochemical profile of mine drainage water

Abiotic data were determined to monitor the chemical characteristics of Mária mine drainage. The temperature of the water sample was 14 °C, with a pH of 6.9 and a conductivity of 1,019 μS cm^−1^, respectively. The water sample contained concentrations of metals, metalloids, and ions, as presented in Table 1.

**Table 1:** Chemical composition of the mine water drainage.

Element	Concentration (mg L^−1^)
SO_4_^2−^	402.35
Mg^2+^	80.9
Ca^2+^	80.17
Cl^−^	35.86
Na^+^	31.7
K^+^	5.65
Mn	5.57
Fe	4.65
F^−^	0.39
NH^4+^	0.239
Li^+^	0.0311
As	0.0104
Ni	0.0091
Co	0.0058
Al	0.0056
Zn	0.0051
Sb	0.0051
Cu	0.0034
Pb	0.0007
Cr	0.0005
Cd	0.0001

### 3.2 Normalization, preprocessing of sequence data and assembly

**Figure 2:**
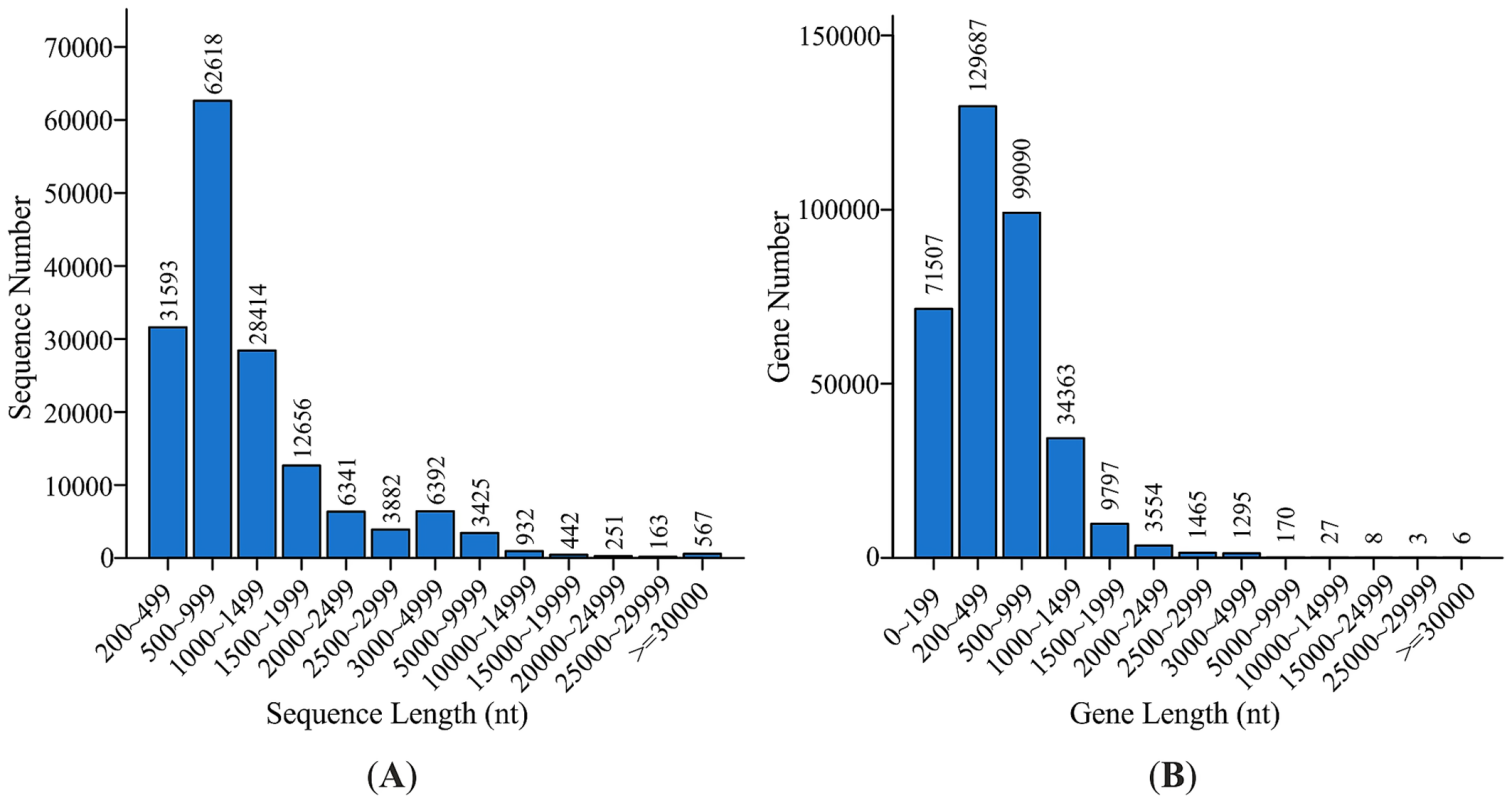
Contig length distribution **(A)** and gene catalogue **(B)**.

The extracted DNA had a concentration of 20.8 ng μL^−1^ and an 260/280 ration of 1.80. DNBSEQ PE150 generated ~227 M clean reads (97.7% of all reads) with an average Q20 score of ~98.95%. High quality sequences lacked ambiguous base cells or base-calling errors (“N” with maximum 0.12%). The GC percentage of generated reads was ~54%. Moreover, reads showed a low amount of adapter contamination (2.18%). The high-quality short reads were *de novo* assembled into 157,676 contigs (252.9 Mbp, N50 = 2,445 bp). Read mapping back to the assembly achieved a 74% success rate. Assembly statistics for Megahit assembly of the sample are detailed in Table 2, while contig length distribution in Figure 2A. MetaGeneMark predicted 482,345 ORFs on contigs ≥ 200 bp. They were clustered at 95% sequence identity using CD-HIT to yield 378,023 non-redundant genes (of which 350,972 were de novo). Gene length distribution was recorded from 200 to more than 30,000 nt (Figure 2B).

**Table 2:** Statistics of assembly result using Megahit software.

Contig number	Assembly length (bp)	N50 (bp)	N90 (bp)	Max (bp)	Min (bp)	Average size (bp)	Mapping rate (%)
157,676	252,875,799	2,445	647	404,057	300	1,603	73.36

### 3.3 Taxonomic classification

The taxonomic survey of our dataset revealed 4 kingdoms, 69 phyla, 141 classes, 287 orders, 628 families, 2,205 genera, and 8,738 species. This spectrum illustrates a sharply expanding taxonomic hierarchy from broad kingdoms to species-level diversity. Variability in microbial community abundance is one of the key focuses of study. At the phylum level, bacterial sequences dominated, representing 18,049,520 reads (99.62%). While archaeal, eukaryotic and viral sequences accounted for 43,351 (0.25%); 21,867 (0.12%) and 2,794 (0.02%) reads, respectively. At the same time, the first five classes studied together represent more than 96% of the community, with Betaproteobacteria alone comprising approximately two-thirds of all reads (66.59%). The Gammaproteobacteria class represented 20.14%, Alphaproteobacteria 6.06%, while Actinomycetes and Bacilli 2.84 and 0.38%, respectively. This hierarchy underlined the overwhelming dominance of Proteobacterial linages. However, the metagenome also captured the diversity of archaea and fungi. The dataset contained the archaea Halobacteria 0.06% (phylum Euryarchaeota), Nitrososphaeria 0.04% (phylum Thaumarchaeota) and the fungi Sordariomycetes 0.06% (phylum Ascomycota).

**Figure 3:**
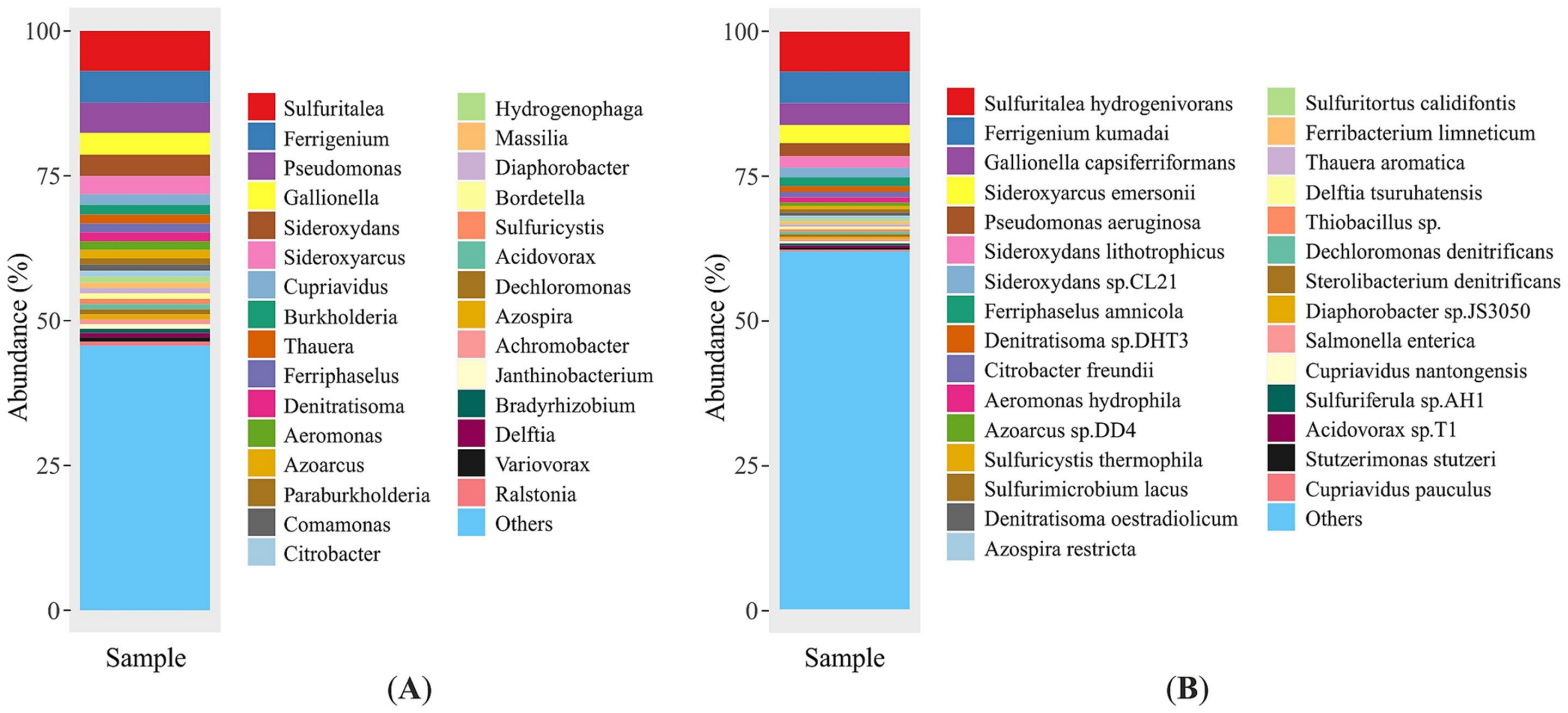
Microbial composition of the environmental sample at genus **(A)** and species **(B)** level, species with abundance less than 0.5 are classified as “others”.

The 30 dominant genera and species, ranking in descending order by read abundance, are plotted in Figure 3. *Sulfuritalea* was found to be the dominant genus in the mine water metagenome, representing 6.93% of the total community (1,255,546 reads). The second most prevalent genus was *Ferrigenium*, representing 5.45% of the community and accounting for 986,339 reads in absolute terms. The genus *Pseudomonas* represents the third most abundant group at this taxonomic level with 5.2% of all sequences (942,319 reads). The following genera were *Gallionella* (3.79%), *Sideroxydans* (3.65%), *Sideroxyarcus* (3.14%), *Cupriavidus* (1.83%), *Burkholderia* (1.67%), *Thauera* (1.54%), *Ferriphaselus* (1.53%) and *Denitratisoma* (1.53%). The abundance of the other bacterial genera was less than 1.5%. While the 30 most abundant genera are shown in Figure 3A, the remaining 2,175 genera, each representing <0.5% of the total community, were grouped as “others”. This category contains only low abundance but taxonomically identified genera.

High-throughput profiling of the mine water metagenome identified eleven taxa that together account for ~30% of all quality-filtered reads. The most abundant species was *Sulfuritalea hydrogenivorans* (1,255,546 reads; 6.93%), followed by the iron-oxidizer *Ferrigenium kumadai* (986,339 reads; 5.45%) and *Gallionella capsiferriformas* (687,130 reads; 3.79%) (Figure 3B). Members of *Sideroxyarcus* lineage were also prominent: *Sideroxyarcus emersonii* (569,891 reads; 3.15%), while *Sideroxydans lithotrophicus* and *Sideroxydans* sp. CL21 contributed 358,632 (1.98%) and 304,010 reads (1.68%), respectively. The facultative anaerobes *Pseudomonas (P.) aeruginosa* (404,803 reads; 2.23%) and *Ferriphaselus amnicola* (278,422 reads; 1.54%) were similarly well represented. Remaining *Pseudomonas* taxa (537,516 reads; 2.97%) were distributed among more than 100 additional species, including *P. fluorescens* (0.16%), *P. putida* (0.12%), *P. chlororaphis* (0.10%), and numerous other low-abundance taxa (<0.1% each). Each of remaining species comprised less than 1% of the total metagenomic reads. Taxonomic classification and corresponding absolute abundance values of the metagenome are provided in Supplementary Table S1.

#### 3.3.1 Functional potential

Functional annotation of the non-redundant gene catalogue against seven databases provided a comprehensive overview of the community’s metabolic and resistance capacities. Statistics of corresponding databases are presented in Table 3, detailed normalized function annotation in Supplementary Table S2 and a summary of functional analysis is available in Supplementary Table S3.

**Table 3:** Statistics of database annotation.

Database	BacMet	Card	Cazy	Cog	Kegg	Nog	SwissProt
All	3,429	102	6,232	118,589	102,113	110,505	66,610

#### 3.3.2 KEGG annotation and analysis profile

**Figure 4:**
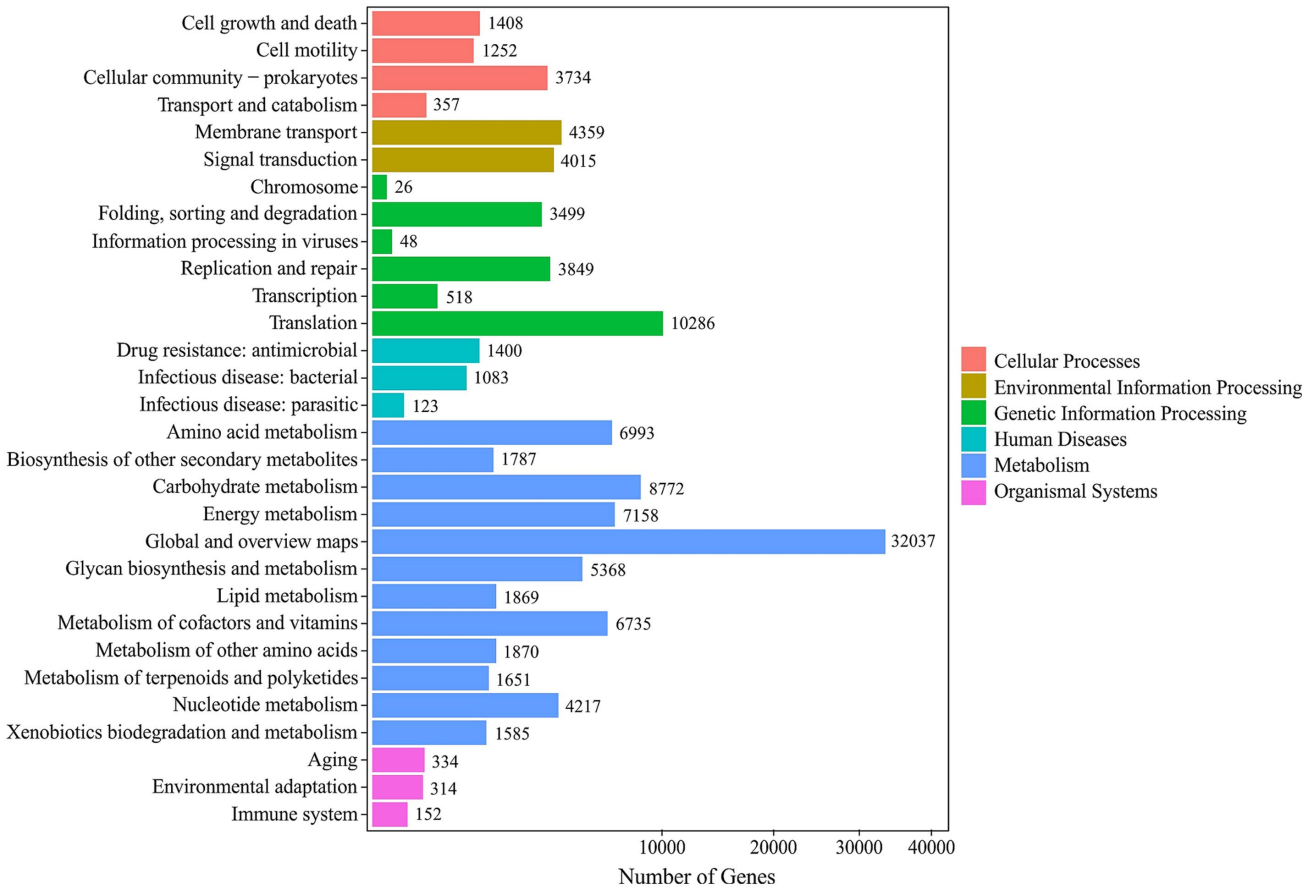
KEGG secondary classification histogram showing the number of genes assigned to each sub-pathway in the Mária mine drainage metagenome. Pathways are grouped by Level 1 category (legend at right).

Functional annotation of the mine water metagenome against the KEGG database assigned a total of 102,113 genes (Figure 4). At KEGG Level 1, “Metabolism” dominated with 601,149 genes, followed by “Genetic Information Processing” (73,352) and “Environmental Information Processing.” Within the broad category of “Metabolism,” key pathways underlying Fe, S, C and N cycling were particularly enriched: “Carbon metabolisms” (57,520 genes), “Energy metabolism” (43,354), and Global and overview maps” (141,606) point to robust primary production and central turnover of carbon. Critically, genes involved in sulfur transformations (“Sulfur metabolism”; 7,496) and nitrogen cycling (“Nitrogen metabolisms”; 2,688) were well represented, as were those enabling autotrophic growth via “Carbon fixation pathways in prokaryotes” (6,885). This functional profile underscores the genetic potential for iron oxidation, sulfur oxidation/reduction, carbon assimilation and nitrogen turnover in the mine drainage community.

KEGG Level 1 functional profiling of the metagenome assigned a total of 98,749,991 annotations across six categories. Metabolisms accounted for 80,772,278 annotations (81.8%), Genetic Information Processing for 6,990,629 (7.1%) and Environmental Information Processing for 4,414,726 (4.5%). At KEGG Level 2, the ten most abundant pathway subgroups comprised 83,814,494 annotations. Global and overview maps dominated with 45,555,676 annotations (54.4%), followed by carbohydrate metabolism (9,065,989; 10.8%) and amino acid metabolism (5,941,847; 7.1%). This profile highlights a metagenome heavily invested in core metabolic pathways and genetic information processing, with secondary emphasis on carbohydrate, energy and amino acid metabolism. These functional distributions reflect the microbial community’s adaptation to the high geochemical stresses of the mine-water environment.

KEGG Orthology (KO) profiling revealed that the most abundant ortholog was K07787 (306,087 counts), encoding the copper/silver efflux system protein (Cus/SilA), which underscores the capacity for heavy metal tolerance (Mealman et al., 2012). The second most prevalent molecular function was K02014 (228,545), an iron-complex outer-membrane receptor involved in siderophore-mediated iron uptake (Stintzi et al., 2000). K00688 (212,157) ranked as the third, glycogen phosphorylase (GlgP), indicating active storage-carbohydrate metabolism (Wilson et al., 2010). Altogether, these KO’s reinforce the focus of the community on metal-stress mitigation and energy-storage strategies. The community’ primary investment in core metabolism and informational functions is presented in the form of KEGG Level 2 circos map (Supplementary Figure S2), while its emphasis on metal efflux and iron uptake is highlighted by the KO circos map (Supplementary Figure S3).

#### 3.3.3 COG/EggNOG annotation and analysis profile

**Figure 5:**
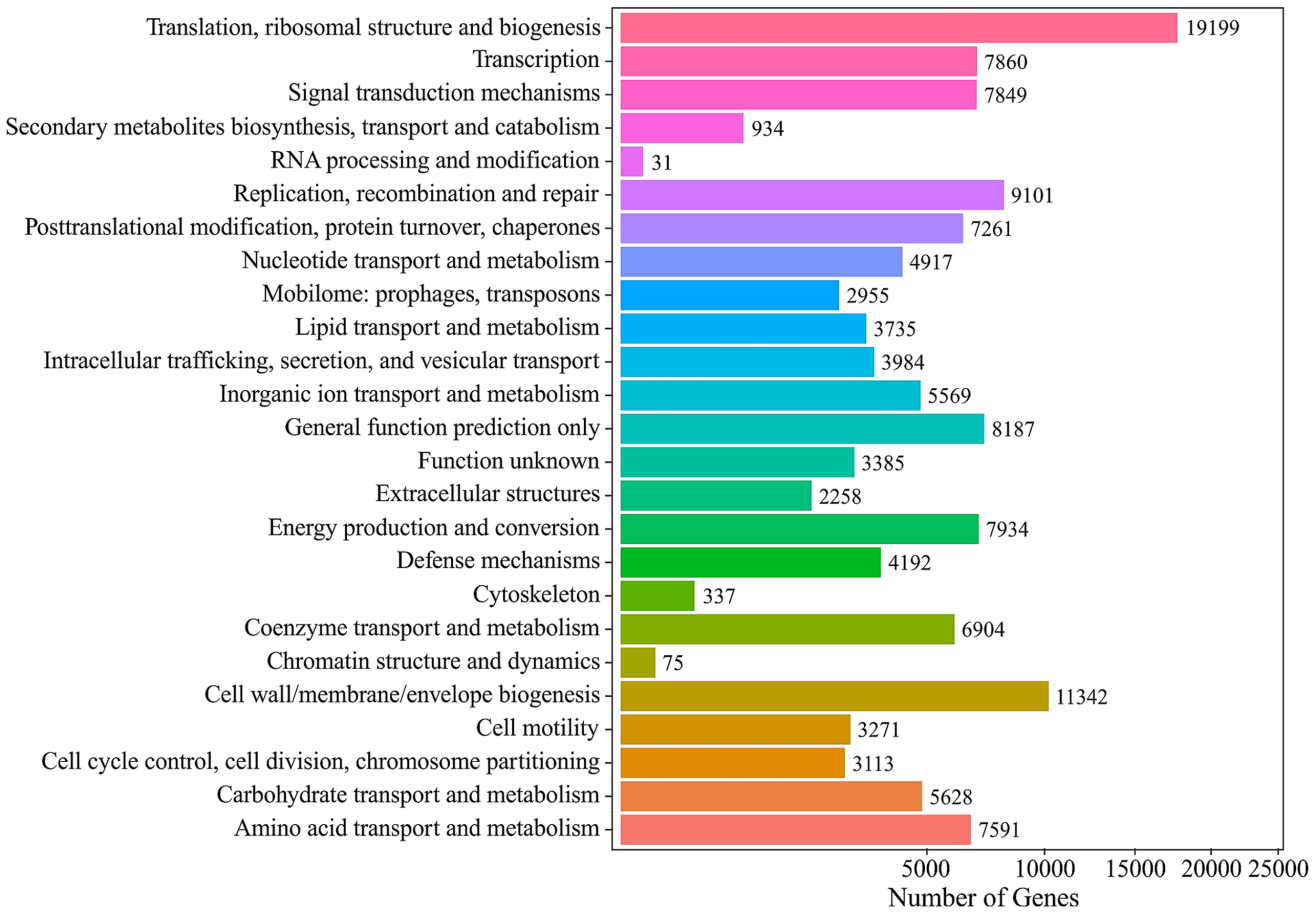
COG annotation histogram of the Mária mine drainage metagenome illustrating the distribution of its genetic content across 24 broad functional categories.

Normalized COG (cluster of orthologous genes) profiling assigned functional categories to all predicted genes, yielding of total of ~659,000 normalized units across 25 categories. The most highly represented functions were “Translation, ribosomal structure and biogenesis” (75,127 normalized units; 11.7%), followed by “Cell wall, membrane and envelope biogenesis” at 50,130 (7.8%), “Energy production and conversion” 44,786 (7.0%) and “Signal transduction mechanisms” 44,190 (6.9%). Notably, the “Function unknown” category accounted for 24,830 units, highlighting a substantial fraction of uncharacterized proteins. Functional annotation against the COG database assigned a total of 137,612 normalized gene hits across 24 functional categories. Gene numbers for every COG functional category are shown in Figure 5. Functional profiling of the mine-water metagenome against the COG database assigned over 50 million gene-hits across 25 broad functional categories. “Signal transduction mechanisms” were the most highly represented category, with 6,855,748 hits, followed closely by “Cell wall, membrane and envelope biogenesis” (6,608,621 hits).

EggNOG represents a database of orthology relationships, functional annotation and gene evolutionary histories, so it provides accurate function prediction. EggNOG normalized functional profiling revealed that the most abundant NOG groups were “Function unknown” (529,030 annotations), “Translation, ribosomal structure and biogenesis” (294,036), “Energy production and conversion” (221,320). NOG functional profiling revealed that the ten most abundant functional groups collectively comprised ~2.18e+8 counts. The dominant category was “Replication, recombination and repair” (2.71e+7), followed by “Function unknown” (2.7e+7) and “Energy production and conversion” (2.6e+7). The EggNog annotation histogram is available as Supplementary Figure S4.

Together, the COG and Eggnog profiles map a microbiome with a high proportion of essential information mechanisms, while the Unknown fraction highlights the potential for uncovering novel functional capabilities.

#### 3.3.4 CAZy annotation and analysis profile

**Figure 6:**
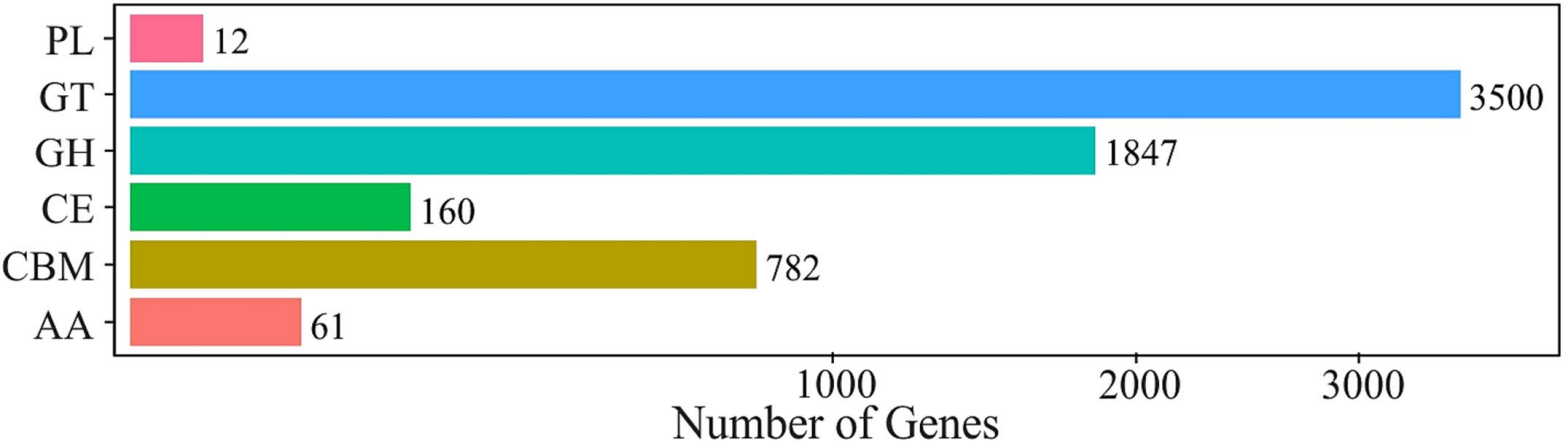
CAZy annotation histogram showing distribution of carbohydraze-active enzyme genes in the metagenome across six classes: glycoside hydrolases (GH), carbohydrate esterases (CE), polysaccharide lyases (PL) and glycosyltransferases (GT), a carbohydrate binding module (CBM), auxiliary activities (AA).

Carbohydrate-active enzyme (Cazy) database contains information on enzymes involved in breakdown, modification and synthesis of glycosidic bonds (Cantarel et al., 2008). CAZy annotation (Level 1) identified a total of 23,089 CAZy distributed across six enzyme classes. Gene numbers of individual enzyme classes are presented in Figure 6.

The most abundant class was glycosyltransferases (GT) with 13,140 hits, followed by glycoside hydrolases (GH) at 9,307. Within the CAZy Level 2 classification, the GT2 family of glycosyltransferases had the highest representation with 4,495. On level 3, one of the most prevalent proteins was BAN36688.1 (399), annotated as a member of the GT2 family, which was identified in sulfur oxidizer *Sulfuricella denitrificans* skB26 (Watanabe et al., 2014). Also determined within this family was protein ADE11609.1 (165), identified in the iron- and sulfur-oxidizing neutrophile *Sideroxydans lithotrophicus* ES-1. A membrane protein BBJ00544 (191) was identified in the microaerophilic iron-oxidizing bacterium *Ferrigenium kumadai* (Watanabe et al., 2021). High prevalences were also determined by AIA01457.1 (407) elongation factor Tu identified in *Streptomyces noursei*, followed by QTH47841.1 (392), identified as 50S ribosomal protein L22 in *Streptococcus zhangguiae* and CUU39686.1 (297) as GDP-mannose 4,6-dehydratase, which was detected in *Helicobacter typhlonius*.

Functional analysis of the non-redundant gene catalogue against the CAZy database at Level 1 revealed a clear dominance of GT (2,187,772) with 39.4% of all CAZy annotations, followed by GH (1,836,358 hits; 33.1%), carbohydrate binding module (CBM; 555,568; 10%), carbohydrate esterases (CE; 91,460, 1.6%), auxiliary activities (AA; 38,282; 0.7%) and polysaccharide lyases (PL; 6,412; 0.1%).

The prevailing dominance of GT and GH enzyme classes indicates high polysaccharide biosynthesis and hydrolysis, reflecting the high dynamic carbon turnover in the Mária mine water microbiome.

#### 3.3.5 Swiss-Prot annotation and analysis profile

In a protein sequence database (Swiss-Prot) annotation, the one of the *de novo* genes yielded its top hit to UniProt accession Q9I310, annotated as bifunctional diguanylate cyclase/cyclic di-GMP phosphodiesterase MucR protein (PA1727, *mucR* gene) from *P. aeruginosa* (strain ATCC 15692). This match was highly significant, with a bit score of 45.9 and e-value of 2.5e-105. Notably, Q9I310 was also the best hit for multiple other sequences in our dataset, suggesting fragmentation of the same gene during assembly or the presence of closely related sequence variants. The second most abundant match was to the cation efflux system protein CusA (P38054, *cusA* gene) characterized in *Escherichia coli* (strain K12), which is a part of a cation efflux system that mediates resistance to copper and silver and thus plays an important role in copper tolerance under anaerobic growth and extreme copper stress during aerobic growth. The next most prevalent protein identified in *Alcaligenes* sp. (strain CT14) was the cation efflux system protein CzcA (P94177, *czcA* gene), with a low cation transport activity for cobalt, and it is essential for the expression of cobalt, zinc, and cadmium resistance.

### 3.4 Resistome

**Figure 7:**
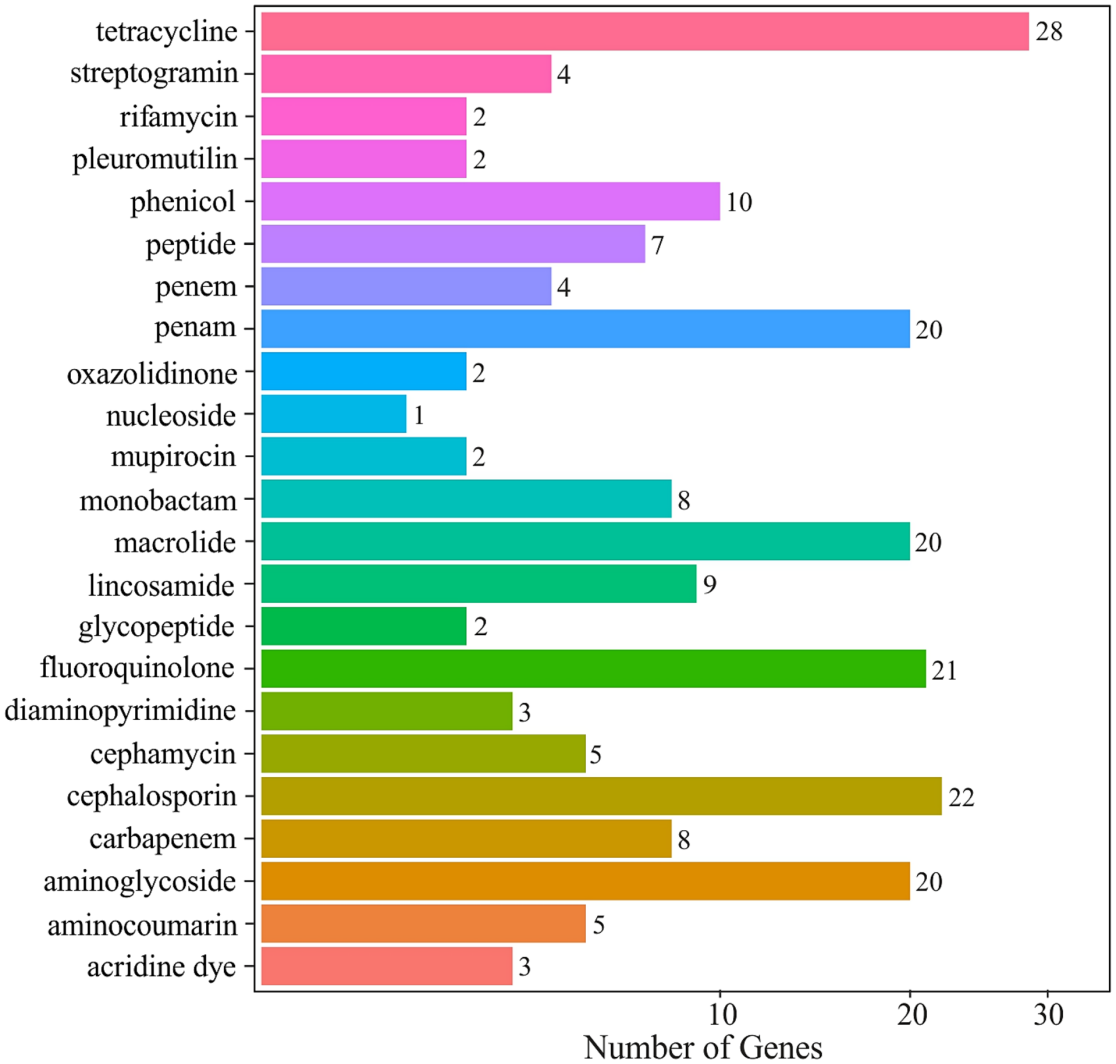
Antibiotic-resistance gene abundance in the mine water metagenome, annotated against the CARD database.

The resistome is composed of genes of different phylogenetic origin, such as antibiotic resistance genes (ARGs) and metal resistance genes (MGRs) (Gillieatt and Coleman, 2024). The normalized CARD profiling of the mine water metagenome exposed a rich and diverse antibiotic resistome. Tetracycline-resistance genes were most abundant, reaching 241.4 normalized hits (28 resistance genes). These included major facilitator superfamily (MFS) tetracycline efflux pumps Tet(B) and Tet(E), as well as ribosomal protection proteins Otr(A) and Tet(32). The ATP-binding cassette (ABC) antibiotic efflux pump Tet(A) also featured prominently. Another widespread gene was *adeF* encoding resistance-nodulation-cell division (RND) antibiotic efflux pump and provides resistance not only to tetracycline but also to fluoroquinolone. The next most abundant classes were fluoroquinolone (226.2 hits), and *β*-lactam resistances were also abundant: cephalosporin (95.7 hits) and penam (93.4 hits). The distribution of all antibiotic-resistance classes is presented in Figure 7. Altogether, annotation against the CARD database identified ~ 193,300 resistance gene hits, of which tetracycline (94,017 hits) and fluoroquinolone (93,923 hits) alone accounted for over 95% of all detected resistance annotations.

**Figure 8:**
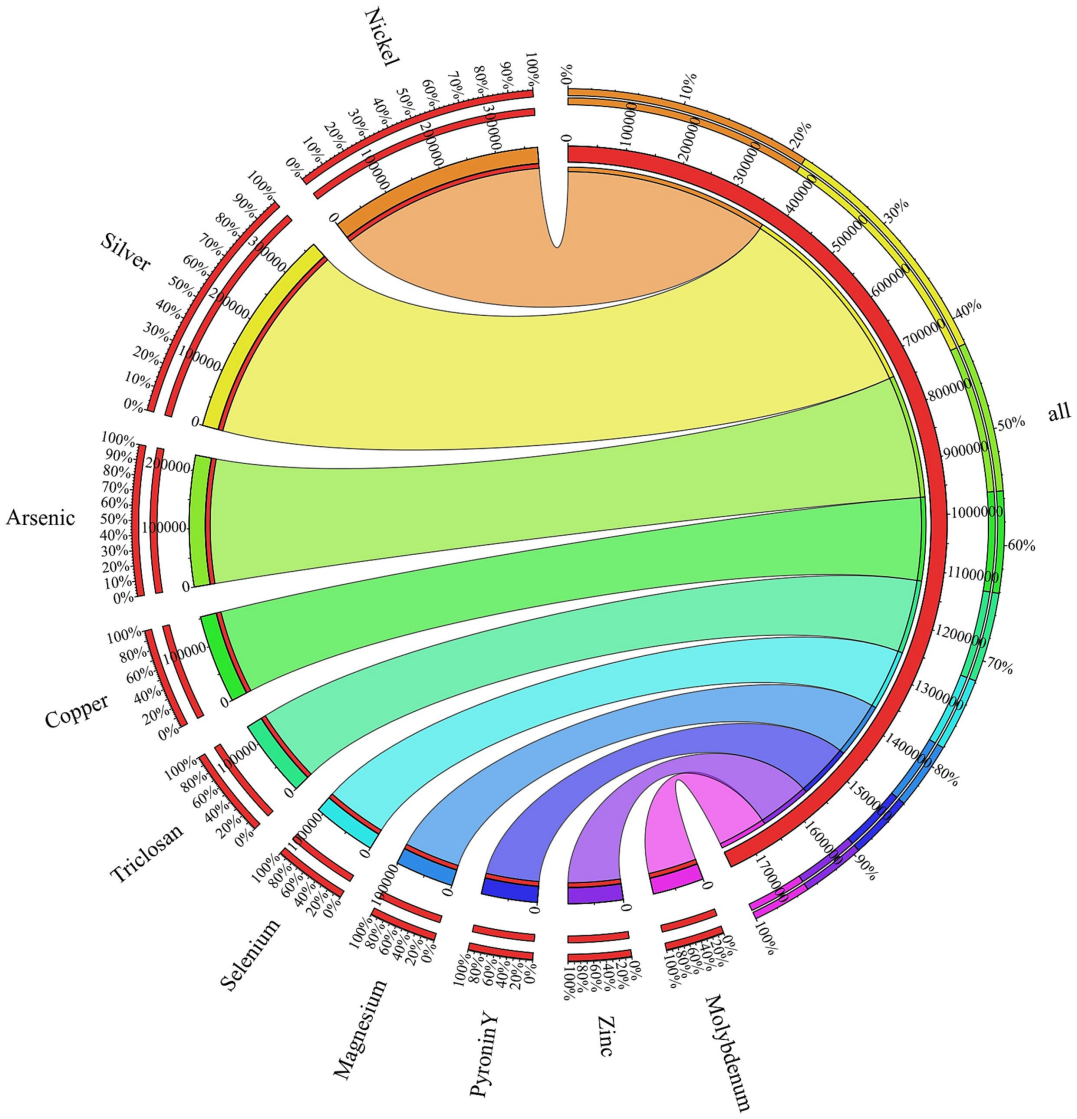
Circos diagram of resistance-associated genes. The outermost ring displays the relative abundance of each resistance class, with colored arc segments corresponding to individual metals or biocides, scaled to the percentage of total hits. Inner ring shows the absolute pool of resistance genes, with arc lengths proportional to the number of hints. Ribbons connect each resistance class (outer arc) to its total count (inner arc), and ribbon widths are scaled to hit number.

Functional annotation of the metagenome against BacMet database revealed a total of 1,530,231 resistance-associated gene hits. The resistance genes were dominated by nickel (372,308 hits) and silver (368,395 hits). This was followed by arsenic (226,740), copper (152,900), trislocan (134,774), selenium (110,557), magnesium (101,209), pyronin Y (97,113), zinc (95,113) and molybdenum (88,635). BacMet-based profiling of the metagenome revealed a set of efflux systems derived from *Ralstonia metallidurans* (strain ATCC 43123) that underlie cobalt, nickel, zinc and cadmium tolerance. The Cation Diffusion Facilitator CnrT (BAC0074) is involved in Co^2+^, Ni^2+^ resistance, while CzcD (BAC0122) decreases the cytoplasmatic concentration of Zn^2+^ and Cd^2+^ cations. Together with CzcB, a periplasmatic membrane fusion protein that bridges inner and outer cell membranes, and the basic inner membrane transport protein CzcA (BAC0119), these components form a multilayered efflux network that enables efficient detoxication of Co^2+^, Ni^2+^, Zn^2+^ and Cd^2+^ under extreme metal stress (Nies, 2003). The cadmium-zinc-nickel resistance protein CznA (BAC0127) derived from *Helicobacter mustelae* (strain ATCC 43772) was also detected. BacMet annotation identified arsenic-related proteins such as a periplasmic oxyanion-binding protein AioX/AoxX (BAC0024) from *Agrobacterium tumefaciens*, that is involved in regulating arsenite oxidation. The *aioX* gene encodes this periplasmatic AsIII-binding protein and is specifically upregulated in response to arsenite exposure (Liu et al., 2012). Similarly, several ORFs matched BAC0033, the arsenic metallochaperone ArsD for an arsenic detoxification pump found in *Escherichia coli.* The role of this protein is to transfer trivalent metalloids to ArsA, the catalytic subunit of an As(III)/Sb(III) efflux pump, which eventually leads to an increase in the rate of arsenite extrusion. As a result, cells are consequently resistant to environmental concentrations of arsenic (Lin et al., 2006). The circos visualization shows the distribution of the metal- and biocide-resistance genes and is presented in Figure 8.

## 4 Discussion

### 4.1 Geochemical profile

Numerous mine drainages in Slovakia have neutral to slightly alkaline character (Liščák et al., 2019). Among the most critical areas is Smolník, where the drainage water (AMD from shaft Pech) is characterized by a low pH (3–4) and a high concentration of iron (400–500 mg L^−1^), which acidifies the Smolník creek (Balintova et al., 2012). According to the measured physico-chemical parameters and classification framework of mine drainages, the mine effluent from the Mária mine belongs to Class III – neutral and not oxidized (Thisani et al., 2020). In general, the neutral pH of mine effluent can result from low pyrite content limiting acid generation or from carbonate host rock or tailings buffering any produced acidity (Waybrant et al., 2002). As the Strieborná vein system is represented mainly by siderite (FeCO_3_; 65–70%) and the remaining part forms a quartz-polysulfidic association, we assume that this is one of the reasons for the formation of the pH neutral mine effluent (Jakubiak, 2008). Monitoring of the geochemical parameters is essential to quickly identify elevated concentration of heavy metals or to prevent environmental catastrophes, such as the recent one in Nižná Slaná, where the Slaná River was strongly contaminated by extremely high amounts of metals, metalloids and sulfate anions (concentration of dissolved substances ~53 g L^−1^). The approximate quantity of pollutants flowing to the Slaná River reached ~90 t per day (Zeman et al., 2022; Kupka et al., 2022).

### 4.2 Taxonomic profile

Metagenomics, which is capable of identifying total genetic material in organisms, provides environmental microbiologists with greater flexibility to immediately examine the enormous genetic variability of microbial communities that inhabit a particular environment. The classification methods of metagenomics sequencing data can be divided into two categories: one is based on 16S rRNA gene sequences, and the other, which was used in this study, is based on whole-genome sequencing fragments. Moreover, the comparative analysis of 16S rRNA and shotgun sequencing data for the taxonomic characterization of the chicken gut microbiota by Durazzi et al. (2021) showed that shotgun sequencing recovered more information about low-abundance genera when a sufficient number of reads were available for taxonomic profiling (> 500,000 reads). Shotgun metagenomics provides more reliable and comprehensive taxonomic resolution than 16S rRNA V3–V4 sequencing, which often recovers only part of the microbial community. In the present study, the spring sample was analyzed by deep shotgun metagenomic sequencing (~227 M clean reads), which allows both taxonomic and functional characterization. Unlike the 16S amplicon approach, shotgun metagenomics captures a broader spectrum of the community, including non-bacterial taxa, and is less subject to primer bias.

Currently, more than 99% of microorganisms cannot be cultivated under laboratory conditions, whereas these uncultured microorganisms may have great application potential and their metabolites may produce valuable compounds (Handelsman et al., 1998). Moreover, the distribution of different bacterial taxa in relation to the biotic and abiotic characteristics of the environment can provide important information on their basic physiology and function in the ecosystem (Mykrä et al., 2017). Multiple studies have also reported that bacterial communities are sensitive to fluctuations in local environmental parameters (Mykrä et al., 2017; Nie et al., 2016; Liu et al., 2018). Moreover, interactions between members of microbial consortia likely play a critical role in optimizing AMD microbial community activity. For example, a significant symbiosis occurs between heterotrophic and certain autotrophic species: autotrophs may depend on coexisting heterotrophs to remove organic compounds that are toxic to them. Heterotrophic acidophiles are able to utilize organic materials produced by acidophilic autotrophs, thereby maintaining community stability (Das and Mishra, 1996).

In the Mária mine water metagenome, class Betaproteobacteria dominated the bacterial community (over 66% of all reads). The most abundant genus was the sulfur-oxidizing genus *Sulfuritalea* followed by the iron-oxidizing group *Ferrigenium* and the ubiquitous pathogen *Pseudomonas* (Herrmann et al., 2017; Khalifa et al., 2018; Lalucat et al., 2022). These lithotrophic taxa were strongly favored in spring, when both Fe (4.65 mg L^−1^) and sulfate (402 mg L^−1^) were elevated, conductivity was high (1,019 μS cm^−1^), and circumneutral pH (6.9) provided optimal conditions for microaerophilic iron- and sulfur-oxidizers. By contrast, a previous study has shown that autumn mine drainage was less extreme, with moderate conductivity of 586 μS cm^−1^, a lower concentration of iron (1.83 mg L^−1^) and sulfate (211.52 mg L^−1^), while the measured values of temperature and pH remained the same (Hagarová and Kupka, 2025). In this case, microbial diversity was assessed solely by 16S rRNA V3–V4 amplicon sequencing. Therefore, the seasonal differences discussed here should be regarded as indicative trends rather than statistically tested associations with geochemical parameters. Heterotrophic genera such as *Pseudomonas* (30%), Gallionellaceae (unclassified; 8.8%), *Polaromonas* (8%) dominated under these conditions (Hagarová and Kupka, 2025). Such seasonal geochemical changes appear to shape microbial consortia, with spring’s high Fe^2+^/S^2−^ availability supporting Fe(II)- and sulfur oxidizers. On the other hand, autumn’s moderate metal-enriched environment seems to advance versatile heterotrophic taxa capable of tolerating relatively low metal stress. Similar seasonal patterns have been reported in other mine drainage systems (McGinness and Johnson, 1993).

The most prevalent bacterial species detected was *Sulfuritalea hydrogenivorans.* This facultative anerobic chemolithoautotroph is able to oxidize thiosulfate, elemental sulfur and hydrogen as sole energy for autotrophic growth. The strain sk43H(T) isolated from a freshwater lake can grow at a temperature of 8–32 °C and an optimum pH of 6.7–6.9 (Kojima and Fukui, 2011). Comparative genomics has also shown that this bacterial species, together with *Sideroxydans lithotrophicus* ES-1, share the oxidation of inorganic sulfur compound pathway, which consists of qr., SoxEF, SoxXYZAB, Dsr proteins, AprBA, Sat, and SoeABC (33). Unlike most neutrophilic Fe(II)-oxidizers, *Sideroxydans lithotrophicus* ES-1 grows autotrophically either by Fe(II) oxidation or by thiosulfate oxidation as its energy source (Zhou et al., 2022). Moreover, *Sideroxydans* sp. CL21 possess organotrophic and also iron-oxidizing capabilities (Hoover et al., 2025). The physicochemical conditions at the sampling site were also suitable for the growth of the neutrophilic, microaerobic iron- and thiosulfate oxidizing chemolithoautotroph, *Sideroxyarcus emersonii* (Kato et al., 2022).

Iron-oxidizing microorganisms have a special ability to catalyze the dissimilatory oxidation of Fe^2+^ to Fe^3+^ playing a key role in the geochemical cycle and in biohydrometallurgical processes for metal extraction (Roberto and Schippers, 2022). Currently, this bacterial group is divided into four various physiological groups (acidophilic, aerobic; neutrophilic, aerobic; neutrophilic, nitrate-dependent anaerobic; anaerobic, photosynthetic group), with most of the neutrophilic, aerobic iron-oxidizers belonging to the class Betaproteobacteria (Hedrich et al., 2011). In our study, the most abundant iron-oxidizing microaerophilic neutrophilic species was represented by *Ferrigenium kumadai* (Khalifa et al., 2018). Strain An22T grows microaerobically and autotrophically, over a temperature range of 12–37 °C and pH 5.2–6.8, not forming extracellular stalks, a characteristic that may contribute to its abundance in the iron-rich mine impacted flows (Khalifa et al., 2018). However, for biohydrometallurgical processes, iron-oxidizing acidophiles capable of enduring extreme conditions such as low pH and high concentrations of metals and metalloids are of most interest (Hedrich et al., 2011). In this study, iron-oxidizing acidophiles formed only a minor fraction of the microbial community, with *Thiobacillus* sp. at 0.43%, *Ferrovum myxofaciens* at 0.16% and *Acidithiobacillus ferrivorans* at 0.11%. *Leptospirillum ferrooxidans* and *Acidiphilium multivorum* were present at even lower abundances. In contrast, the aerobic thermoacidophilic crenarchaeon *Sulfolobus acidocaldarius* accounted for 6.07% (Chen et al., 2005).

*Gallionella* spp. have been found to be dominant in various mine environments, and they play an important role in AMD generation (Bruneel et al., 2006; Hallberg et al., 2006; He et al., 2007). For example, *Gallionella capsiferriformans* is a microaerobic neutrophilic iron-oxidizer. It typically inhabits a transition zone from microoxic groundwater to oxic surface water with a high heavy metal concentration (especially ferrous iron), moderate temperature (13 to 15 °C) and a circumneutral pH (Fabisch et al., 2016; Lin et al., 2012). These parameters were also typical for the selected sampling site between the Mária mine drainage and the Slaná River.

Genus *Pseudomonas* is widely distributed, plays relevant ecological roles and is able to adapt to fluctuating environmental conditions (Silby et al., 2011). The most abundant species of this genus in our mine water metagenome was the species *P. aeruginosa* (class Gammaproteobacteria, with 20% of all reads). It was found that *P. aeruginosa* displayed no significant growth inhibition in treated mine water containing 1,890 mg L^−1^ SO_4_^2−^, while *Escherichia coli* was significantly sensitive under the same conditions (Stoica et al., 2022). This finding highlights the exceptional tolerance of *P. aeruginosa* to extreme sulfate stress, underscoring its potential dominance in the high-sulfate mine water environment. This high tolerance probably explains the marked relative abundance in the Mária mine drainage. Moreover, genus *Pseudomonas* contains more than 25 species associated with opportunistic human infection, including *P. aeruginosa*, which can be found in most moist environments (Baron, 1996). Such infections are mainly challenging because of the organisms’ broad intrinsic antimicrobial resistance, largely mediated by multidrug efflux pumps and permeability barriers (Lister et al., 2009). Specifically, members of the Resistance Nodulation Division (RND) family seem to be the most significant contributors to its antimicrobial resistance. Efflux systems such as MexAB-OprM, MexCD-OprJ and MexXY-OprM accommodate and provide resistance to *β*-Lactams, which are commonly used in the treatment of *P. aeruginosa* infections (Paul et al., 2010; Poole, 2004). Importantly, several of these efflux systems (e.g., CzcCBA) also confer resistance to heavy metals, providing cross-protection in metal-rich environments (Perron et al., 2004). Such a high resistance potential raises concerns that metal-rich mine waters may serve as a reservoir where antibiotic and metal resistance determinants co-occur and persist (Seiler and Berendonk, 2012; Fawwaz Alfarras et al., 2022). Consequently, the detection of *P. aeruginosa* in the Mária mine drainage is both ecological and clinically relevant. It underscores the adaptive success of this bacterium to metal-rich environments and its possible role in disseminating resistance determinants across environmental and human-associated microbiomes. The second most common species of the genus *Pseudomonas* was *P. fluorescens*, an environmental bacterium primarily associated with soil and rhizosphere health (Taylor et al., 2025). While not typically pathogenic to humans, it is occasionally detected at low abundance in the native microbiota and possesses functional traits that enable survival in mammalian hosts (Scales et al., 2014). On the other hand, *P. putida* represents a bacterium living in water and soil, characterized by physiological robustness, metabolic versatility, and high tolerance to stress. Furthermore, some strains, such as *P. putida* KT2440 and mt-2, have proven valuable in bioremediation efforts due to their ability to degrade aromatic compounds such as toluene and xylenes (Volke et al., 2020).

Genus *Legionella* represents aerobic chemoorganotrophic, gram-negative bacilli. *Legionella* spp. can be found in surface water, ponds, streams, soils, and man-made aqueous environments such as conditioning cooling towers, and the optimum pH for their growth is 6.8–7 (Steinert et al., 2002). The most abundant species of this genus were *Legionella* sp. MW5194 and *Legionella pneumophila*, however their abundance was less than 0.23e-3% despite the optimal pH of the mine water for their growth.

### 4.3 Functional profile

The mine water metagenome can be utilized not only to evaluate the structure and diversity of the mine water community but also to identify meaningful microbial genera and species that drive geochemical transformation of iron, sulfur, carbon and nitrogen. In the recent study, Grettenberger and Hamilton reported 29 novel metagenome-assembled genomes from an acid mine drainage site. The genomes span 11 bacterial phyla and one archaeal phylum and revealed key metabolic functions including Cyc2-like cytochromes for Fe(II) oxidation, Sox and Dsr pathways for sulfur cycling and RubisCO-mediated carbon fixation (Grettenberger and Hamilton, 2021). Understanding these metabolic pathways is critical for advancing and optimizing applied biotechnological processes in AMD remediation and raw material recovery. For example, bioleaching test of tetrahedrite concentrate from the same Mária mine, whose water metagenome we have profiled, yielded over 4 g L^−1^ Cu (> 80%) after 120 days using iron-oxidizing acidophiles from the cultures collection (DSMZ) (Kupka et al., 2025).

In general, metals are toxic by a wide range of mechanisms. For example, bacterial cells are effectively killed upon contact with copper surfaces by the copper ions released (Espírito Santo et al., 2011). On the other hand, silver ions harm cells by the inappropriate binding to biomolecules and shut down essential functions such as the respiratory electron transport chain or DNA replication (Holt and Bard, 2005). Metal resistance can be achieved by various strategies such as enzymatic detoxication, intra- and extracellular chelation, or transport systems, whereas transport systems are widespread in bacterial cells and serve to limit metal concentrations by removing of metals from cells (von Rozycki and Nies, 2009). *Cupriavidus metallidurans* CH34 is a model organism for heavy metal detoxification and a variety of biotechnological applications. In this work, 21 different bacterial species of the genus *Cupriavidus* were identified with low prevalence (below 0.01%). For instance, the species *Cupriavidus necator* has been identified, along with the *hoxH* gene encoding the high-affinity nickel transporter HoxN (NiCo transporter family) responsible for nickel uptake.

The presented resistome profile closely mirrors the geochemical composition of the mine drainage and its surrounding host rock, particularly the tetrahedrite rich Strieborná vein. The Strieborná vein is characterized by relatively high Ag content (up to 1 wt%) (76), significant Cu (40–46 wt%) and Sb (26 wt%) content (Hagarova et al., 2025). Alongside a substantial level of Mg^2+^, As, Ni and Zn in the mine water, it illustrates how the availability of these metals and metalloids drives co-selection of resistance genes. Moreover, co-selective pressure from heavy metals has been shown to promote the persistence and dissemination of not only metal but also antibiotic resistance determinants across environmental bacterial communities (Gillieatt and Coleman, 2024; Larsson and Flach, 2022). Due to agricultural and aquacultural practices, antibiotics are transferred to soil and water environments (Heuer et al., 2011). When antibiotics enter the soil environment, they can leach into the aquatic environment (Boxall et al., 2002). Together with heavy metals, they can drive co-selection and selection towards antibiotic resistant bacteria (Seiler and Berendonk, 2012). Antibiotics such as tetracyclines and fluoroquinolones persist in the environment for longer periods of time, spreading more and accumulating at higher concentrations than, for example compared to penicillin (Giedraitienė et al., 2011). Previous studies have shown that Zn directly triggers the selection of tetracycline resistance genes, such as *tet*A, *tet*C and *tet*G genes (Palm et al., 2008; Yamaguchi et al., 1990), which function as efflux pump. Additionally, *tet*W and *tet*B/P genes encoding ribosomal protection proteins that are detected in metal contaminated areas (Knapp et al., 2017; Chen et al., 2019). The presented enrichment of tetracycline- and fluoroquinolone-resistance markers in the mine water resistome thus reflects both their environmental persistence and co-selective pressure exerted by heavy metals.

Functional profiling of the Mária mine water revealed the pronounced representation of genes involved in sulfur, iron, carbon and nitrogen metabolisms. Such gene enrichment mirrors microbial adaptation to the site’s metal-rich chemistry. These geochemical profiles are characteristic for mine drainage environments and create strong selective pressure for chemolithotrophic Fe(II)- and S-oxidizers. Mapping of the non-redundant gene catalogue against the KEGG Orthology (KO) database, which links genomes to biological systems by functional orthologs, recovered signatures for geochemical cycling pathways of iron, sulfur, carbon and nitrogen.

The predominant form of iron in the environment under aerobic conditions is the ferric ion. Bacteria and fungi synthetize siderophores, as iron-complexing molecules, that allow cells to selectively chelate Fe(III) and take up iron from its environment (Crichton, 2012). There is a close association between determined the iron complex outer-membrane receptor protein TC.FEV.OM (K02014) and siderophores. It was also confirmed that selected *Pseudomonas* bacteria capable of secreting siderophores can significantly improve the absorption efficiency of Cd in sorghum (Zhou et al., 2021). Considering the sulfur cycle, the basic components of the Sox sulfur-oxidation enzyme system, such as SoxA (K17222; 26.4), SoxX (K17223; 30.3), SoxB (K17224; 14.4), were all clearly detected (Meyer et al., 2007). Meanwhile, the dissimilatory sulfite reductase subunits DrsA (K11180; 213.3) along with DrsB (K11181; 228.3) were also ranked among the most abundant, which indicates strong sulfate-reduction potential. By contrast, sulfide:quinone oxidoreductase Sqr (K17218) showed one of the highest normalized abundance (419.2). This enzyme catalyzes the oxidation of sulfide to sulfur via electron transfer to the membrane quinone pool in microorganisms and mitochondria (Duzs et al., 2021). Moreover, the annotation results recovered the RubisCO large subunit protein RbcL (K01601) at a normalized abundance of 254.8, highlighting the capacity for CO_2_ fixation into organic compounds. Plants rely on one highly conserved so-called green-type enzyme consisting of eight large subunits and eight small subunits (L_8_S_8_). In contrast, proteobacteria often have a variety of different rubiscos, including both red and green form I enzymes and form II rubisco (Andersson and Backlund, 2008; Satagopan et al., 2014; Badger and Bek, 2008). Moreover, chemoautotrophic proteobacteria oxidizing sulfur and other inorganic substances are able to use rubisco to carry out “dark” CO_2_ fixation across environments – from AMD sites to the dark oceans (Swan et al., 2011). The studied metagenome contains a full set of nitrogen-cycling potential. Copper-containing nitrite reductase NirK (K00368; 24.1) has been identified and is known to be used by both bacterial and fungal denitrification to reduce NO_2_^−^ to NO, which is then utilized to produce N_2_O and/or N_2_ with organic carbon as an electron donor (Shoun et al., 2012; Zumft, 1997; Long et al., 2014). Moreover, the presence of nitrogenase components such as nitrogenase molybdenum-iron protein alpha chain NifD (K02586; 29.3), nitrogenase iron protein NifH (K02588; 28.4) and nitrogenase molybdenum-iron protein beta chain NifK (K02591; 29.7) indicated potential for atmospheric N_2_ fixation (Rubio and Ludden, 2005). Also, incorporation of NH_4_^+^ into cellular biomass is catalyzed by glutamine synthetase glnA (K01915; 228.4) and glutamate synthase gltB (K00265; 203.1) (Merrick and Edwards, 1995). This pathway is also known as glutamine synthetase (GS)/ glutamate synthase (GOGAT) cycle, where GS is highly conserved, which aligns with the possibility that it assimilates ammonia in most bacterial taxa (Kumada et al., 1993; Reitzer, 2003). In this study, several genera have been identified that play a dominant role in the nitrogen cycling and underscore a strong genetic potential for both nitrogen removal (via denitrification) and nitrogen input (via N_2_ fixation). The most prevalent genera, such as *Denitratisoma*, *Thauera*, *Azoarcus*, *Azospira*, *Comamonas* and others, collectively accounted for more than 9% of the community. Among these genera, *Thauera* represents a significant genus not only capable of dentification, but can also perform phosphorus removal and sulfide oxidation in S-EBPR (sulfur conversion-associated enhanced biological phosphorus removal) systems, thus making it applicable for remediation of environmental contaminants in wastewater treatment (Zhang et al., 2017).

Comparative use of KEGG and eggNOG annotations strengthens functional interpretation (Zeller and Huson, 2022). KEGG analysis highlighted significant enrichment of genes involved in metabolic pathways (e.g., sulfur oxidation via Sox, carbon oxidation by RubisCO) with precise linking to biogeochemical cycles. By contrast, eggNOG clustered genes into broad ortologous groups (e.g., energy production and conversion, amino acid and carbohydrate transport and metabolism, signal transduction mechanisms). This overlap confirms the dominance of energy and nutrient cycling pathways. At the same time, it illustrates the complementary resolution of these databases. KEGG database provides pathway-oriented detail, whereas eggNOG classifies genes into broader orthologous groups with lower biochemical specificity. Using both databases thus increases the robustness of functional interpretation by combining complementary perspectives.

It is important to emphasize that the present study was designed as a baseline characterization of the current status of the Mária mine drainage. The taxonomic and functional profiles presented here therefore reflect the microbial community structure and geochemical conditions at a single sampling point. The objective of this study provides the first comprehensive description of this unique neutral-pH, metal-rich drainage.

## 5 Conclusion

Given that this mine water drainage is the sole known mine water effluent from the Mária mine, the metagenomic profile revealed a unique baseline of microbial community that is both taxonomically and functionally tailored to high-metal and neutral pH conditions. Betaproteobacteria dominate (> 66%), led by chemolithotrophic genera such as *Sulfuritalea* (6.9%), with the most prevalent bacterial species detected *Sulfuritalea hydrogenivorans*, followed by genera *Ferrigenium*, *Gallionella*, *Sideroxydans*, alongside the heterotroph *Pseudomonas*. Shotgun metagenomics revealed pronounced enrichment of genes for iron cycling (e.g., TC.FEV.OM), sulfur cycling (e.g., *soxA*, *soxB*, *soxX*, *dsrA*, *dsrB*), carbon turnover (GT and GH CAZy families) and nitrogen cycling (*nirK*, *nifH*, *glnA*). The resistome was overwhelmingly composed of tetracycline and fluoroquinolone genes (> 95% of CARD hits) and extensive Ni, Ag, As, Cu and also Zn transport systems (e.g., CnrT, CzcD, CzcA, CznA, ArsD and AioX/AoxX proteins). Together, these findings position this community as a rich source of lithotrophic strains. In particular, neutrophilic Fe(II)-oxidizers emerged as promising targets for future isolation, kinetic characterization and pH tolerance testing to evaluate their potential in biotechnological innovations such as bioremediation and recovery of critical raw materials.

This first integrated dataset establishes an essential reference point for future monitoring. Since this is a single-timepoint survey of the sole Mária mine effluent, future work has to include temporally and spatially replicated sampling in order to capture community dynamics and validate functional resilience. The observed taxonomic and functional patterns therefore do not represent long-term trends, but rather reflect the current state of the microbial community under the prevailing geochemical conditions. These efforts align with EU priorities such as the European Green Deal (European Commission, 2019), Critical Raw Material Resilience: charting a Path towards more excellent Security and Sustainability (European Commission, 2020) and Regulation of The European Parliament and of The Council establishing a framework for ensuring a secure and sustainable supply of critical raw materials and amending Regulations (European Commission, 2023).

## Data Availability

The data presented in the study are deposited in the NCBI repository, accession number PRJNA1297402. Our SRA records will be accessible with the following link: https://www.ncbi.nlm.nih.gov/sra/PRJNA1297402.
