# Supplementary material for: Insights into the microbiome of mine drainage from the Mária mine in Rožňava, Slovakia: a metagenomic approach
Source: Front Microbiol. 2025 Nov 28;16:1675058. doi: 10.3389/fmicb.2025.1675058 (PMC12699271; doi:10.3389/fmicb.2025.1675058)
Supplement: Supplementary file 2 [file Supplementary_file_1.docx]

Supplementary Material


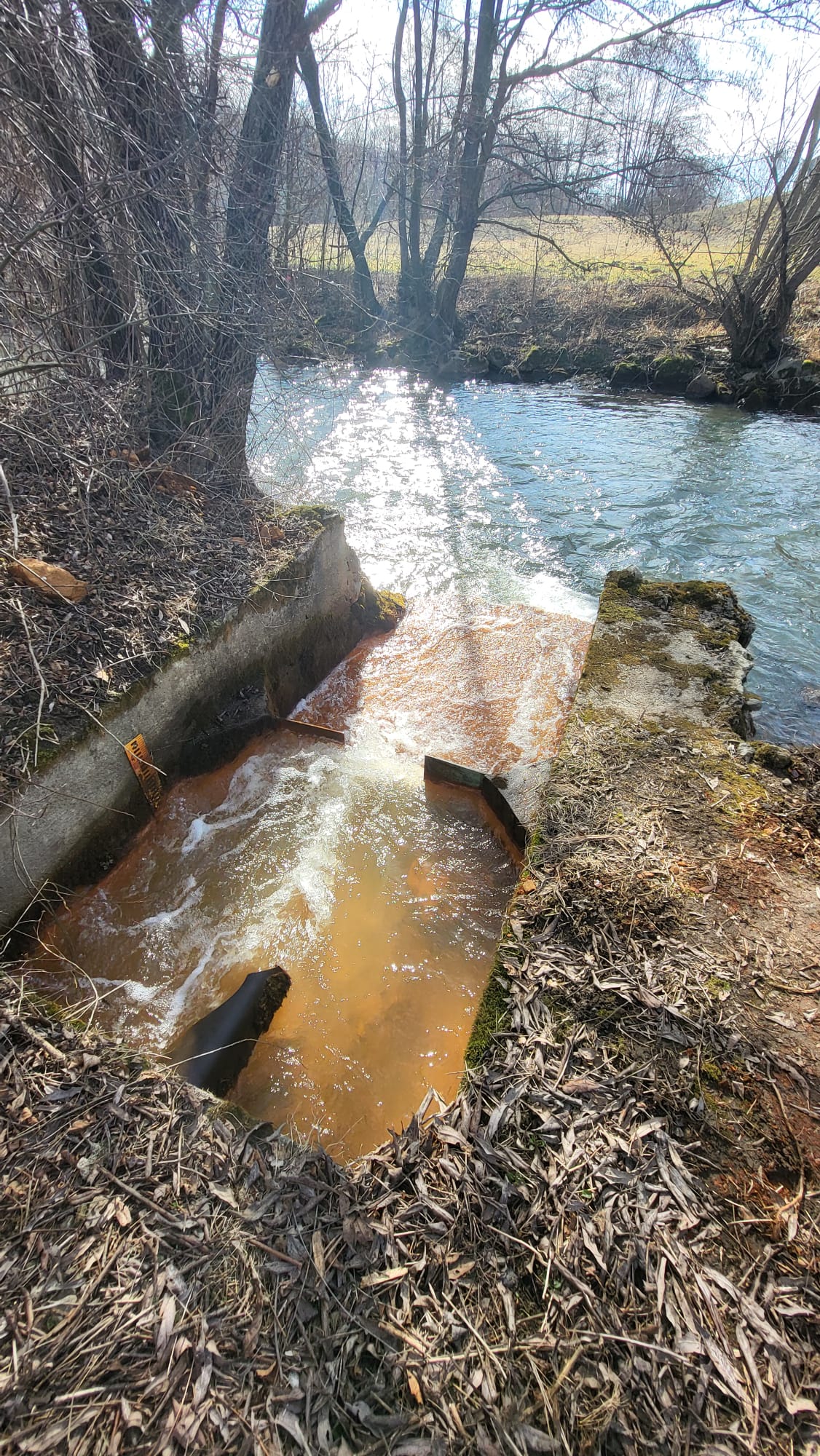


(**A**)


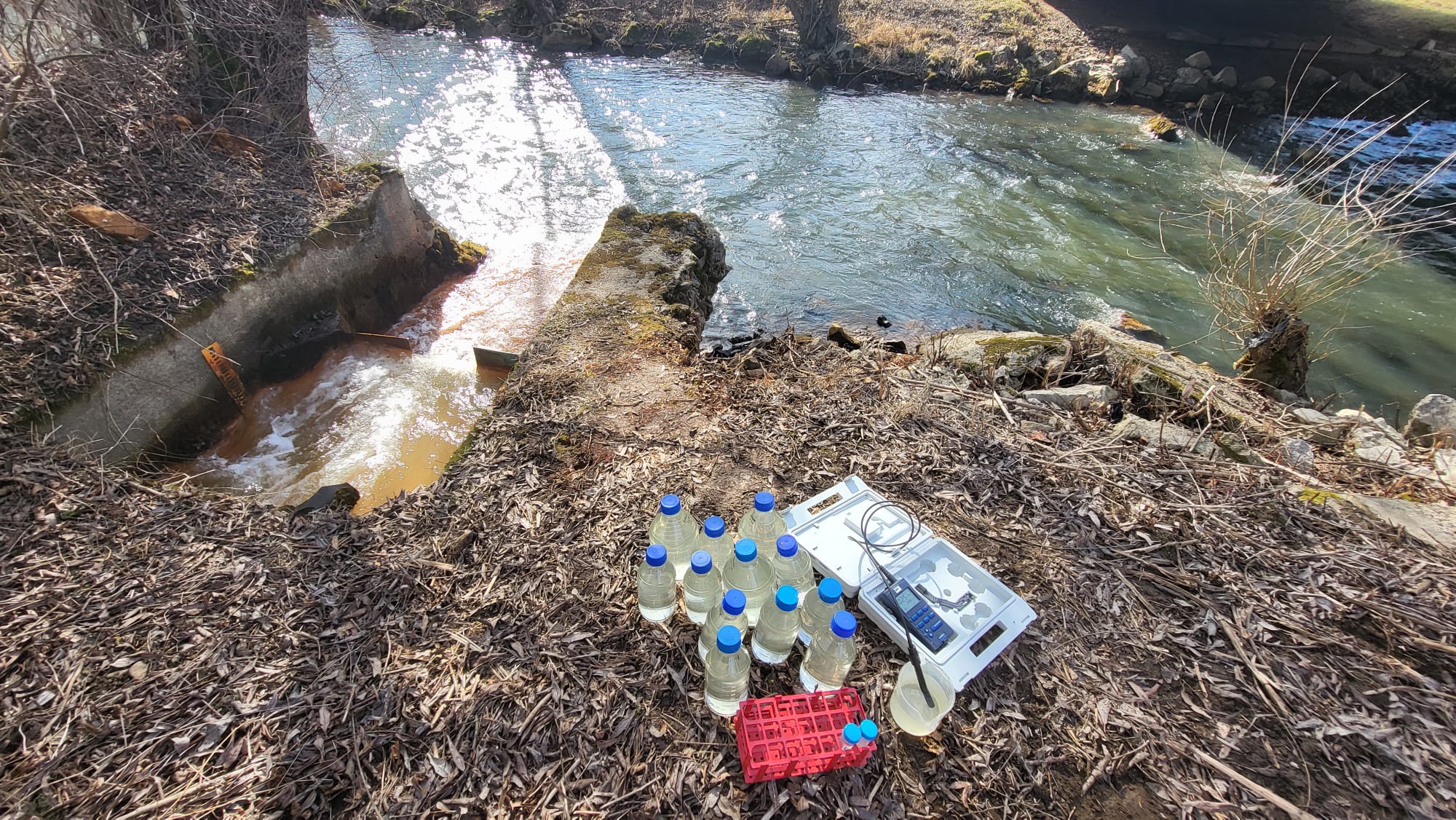


(**B**)

**Supplementary Figure S1.** Photographs (**A**) and (**B**) of the sampling site of the Mária Mine in Rožňava. The site is located in proximity to a local road but outside the residential part of Rožňava. The nearest human habitation is a single-family house, situated at a distance of ~200 m in a straight line from the sampling point and the second family house on the other side of the same local road (distance of ~100 m in a straight line from the sampling point). The area is not subject to animal grazing or significant anthropogenic disturbance.


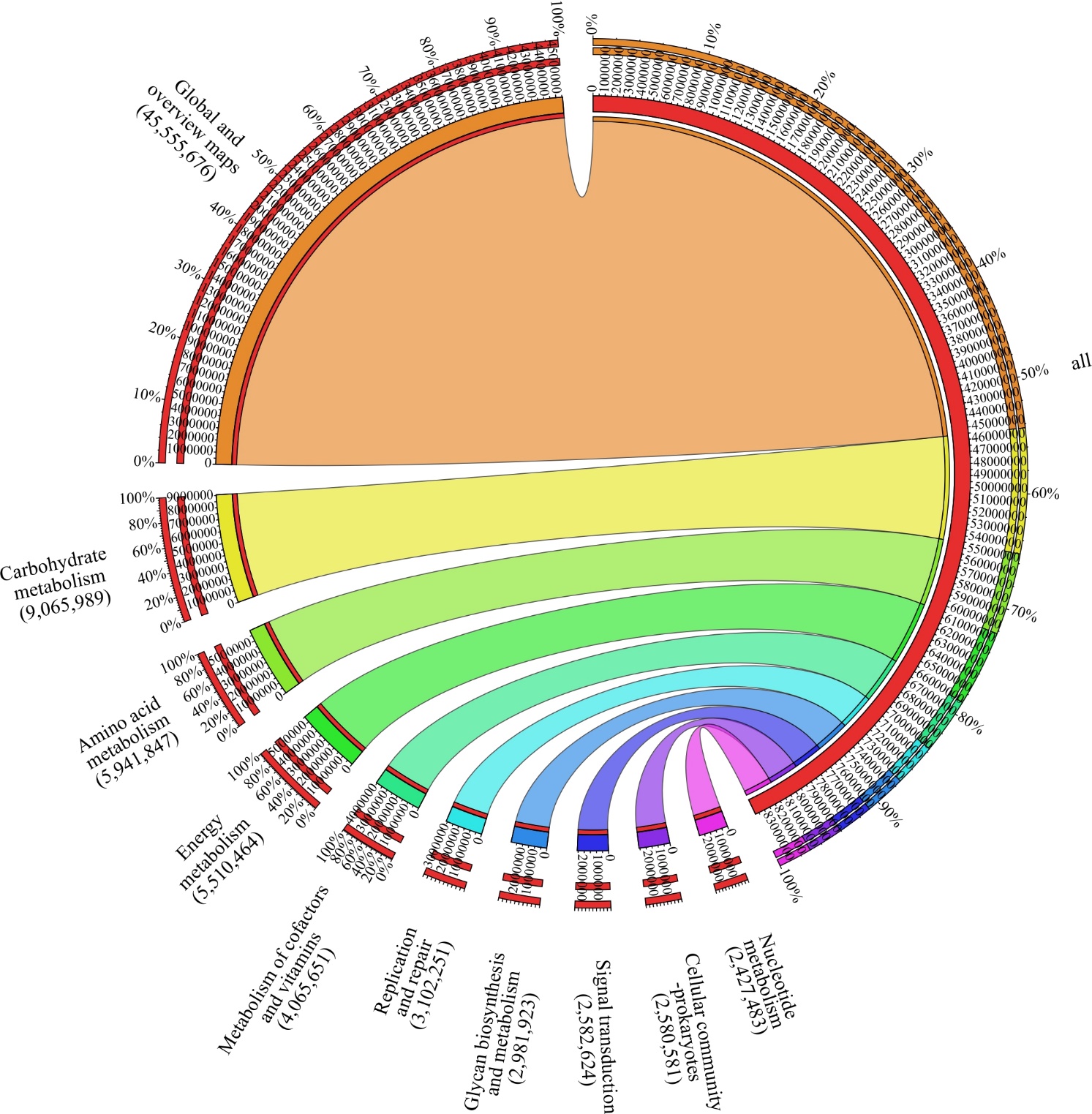


**Supplementary Figure S2.** KEGG Level 2 circos map linking the total annotation pool (outer “all” sector) to their ten most abundant Level 2 sub-pathways in the Mária mine drainage metagenome. Wide inner ribbons trace the flow from the total pool to each Level 2 pathway: global and overview maps dominated (45,555,676 annotations (54.4 %), followed by carbohydrate metabolism (9,065,989; 10.8 %), amino acid metabolism (5,941,847; 7.1 %), Energy metabolism (5,510,464; 6.6%), Metabolism of cofactors and vitamins (4,065,651; 4.9 %), Replication and repair (3,102,251; 3.7 %), Glycan biosynthesis and metabolism (2,981,923; 3.6 %), Signal transduction (2,582,624; 3.1 %), Cellular community-prokaryotes (2,580,581; 3.1 %) and Nucleotide metabolism (2,427,483; 2.9 %).

**
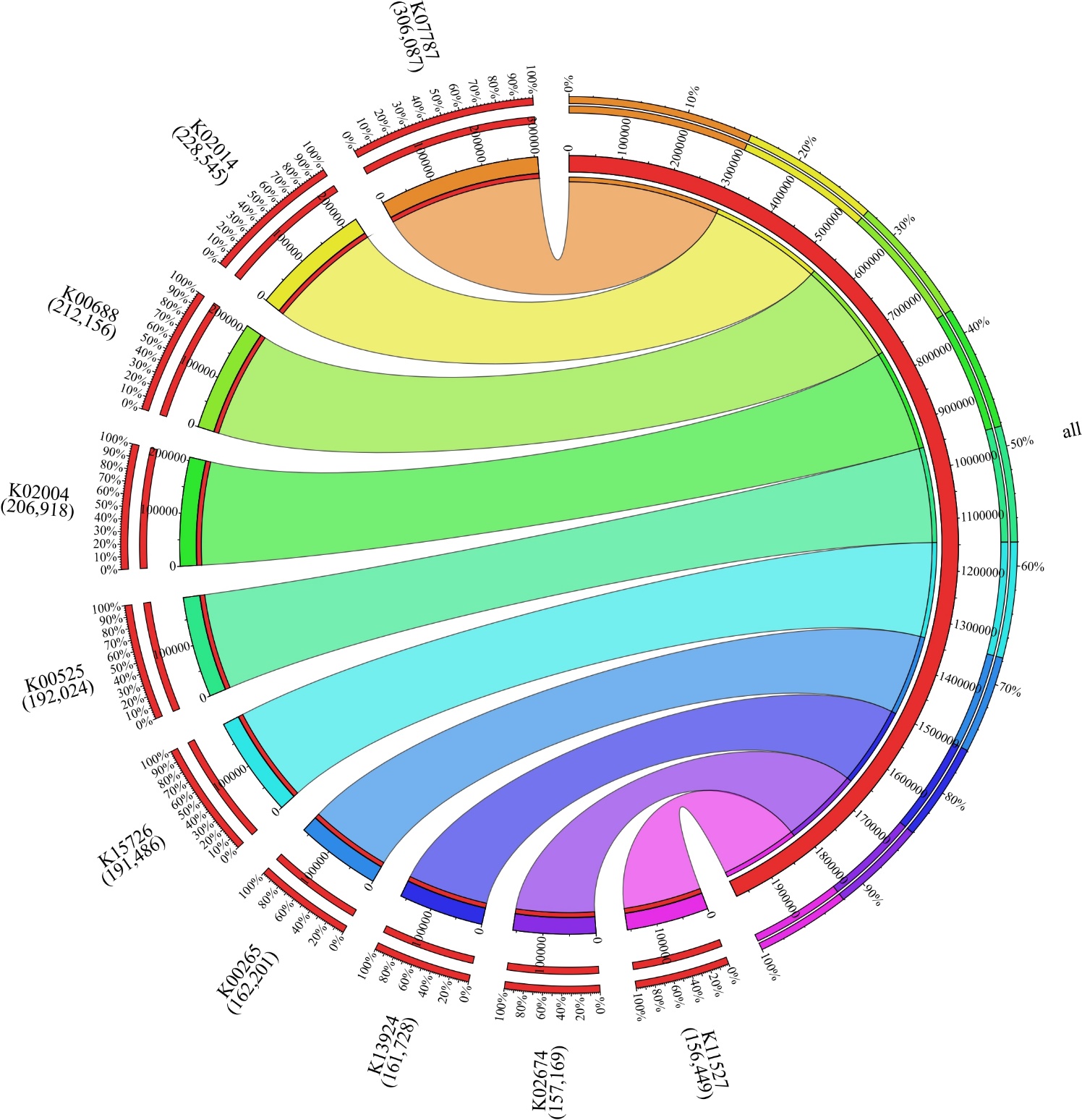
**

**Supplementary Figure S3.** Circos map of the top ten abundant KO groups within the Mária mine drainage metagenome. The outer “all” sector (red) represents the complete KO annotation pool. From this pool, ten ribbons fan inward and indicate the contribution of each orthologue: K07787 encoding the copper/silver efflux system protein (306,087), K02014 (an iron-complex outer-membrane receptor involved in siderophore-mediated iron uptake; 228,545), K00688 (glycogen phosphorylase; 212,156), [K02004](https://www.kegg.jp/entry/K02004)  (putative ABC transport system permease protein; 206,918), [K00525](https://www.kegg.jp/entry/K00525) (nrdA, nrdE; ribonucleoside-diphosphate reductase alpha chain; 192,024), [K15726](https://www.kegg.jp/entry/K15726)  (czcA, cusA, cnrA; heavy metal efflux system protein; 191,486), [K00265](https://www.kegg.jp/entry/K00265) (gltB; glutamate synthase (NADPH) large chain; 162,201), [K13924](https://www.kegg.jp/entry/K13924) (cheBR; two-component system, chemotaxis family, CheB/CheR fusion protein; 161,728), [K02674](https://www.kegg.jp/entry/K02674) (pilY1; type IV pilus assembly protein PilY1; 157,169), [K11527](https://www.kegg.jp/entry/K11527) (two-component system, sensor histidine kinase and response regulator; 156,449).


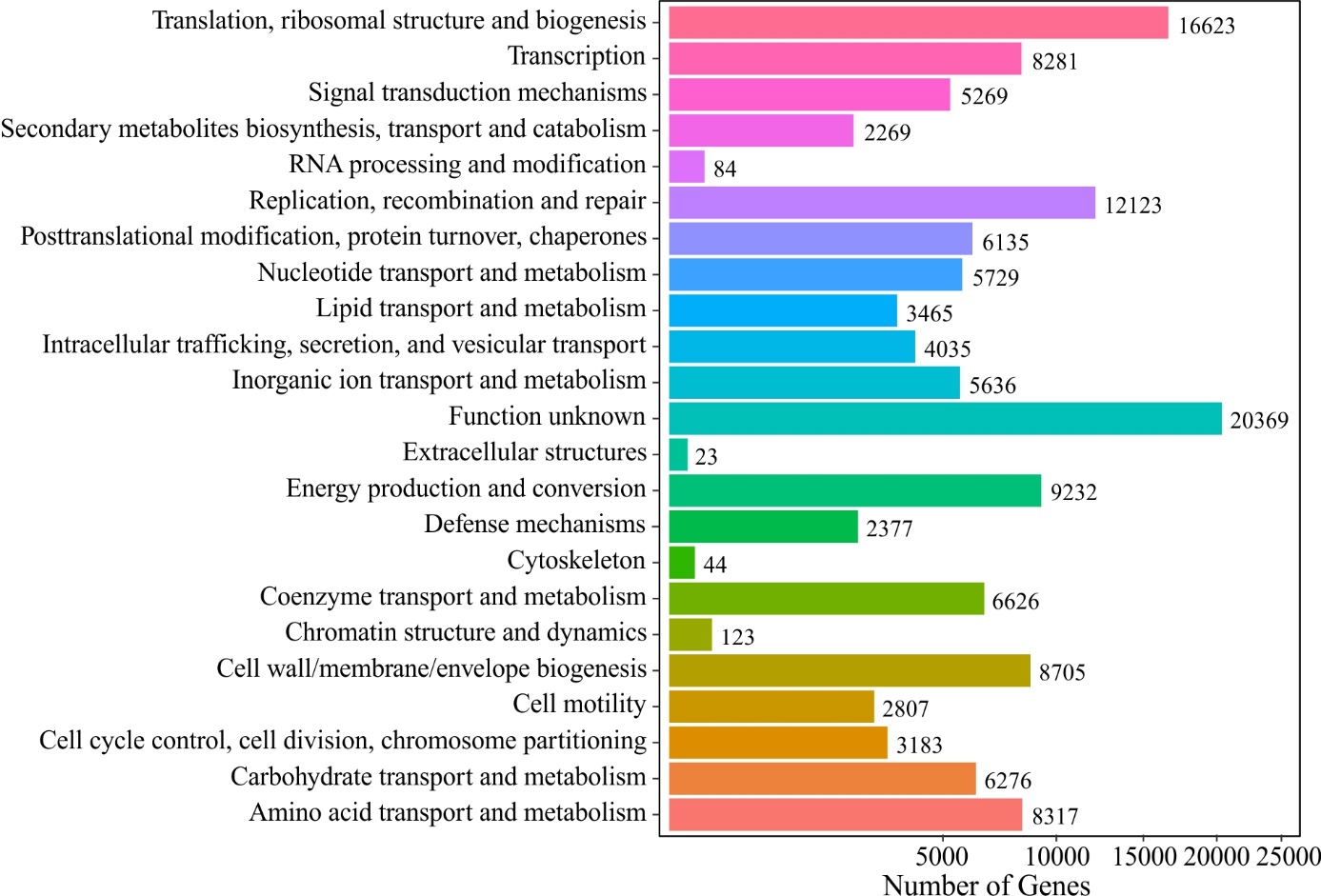


**Supplementary Figure S4.** EggNOG functional classification of the metagenome, showing the number of genes assigned to each NOG category.
